# Anthropogenic Matrices Favor Homogenization of Tree Reproductive Functions in a Highly Fragmented Landscape

**DOI:** 10.1371/journal.pone.0164814

**Published:** 2016-10-19

**Authors:** Magda Silva Carneiro, Caroline Cambraia Furtado Campos, Luiz Alberto Beijo, Flavio Nunes Ramos

**Affiliations:** 1 Instituto de Biologia, Universidade Estadual de Campinas (UNICAMP-SP), Cidade Universitária Zeferino Vaz, Barão Geraldo, Campinas, SP CEP 13083-970, Brazil; 2 Instituto de Ciências da Natureza, Universidade Federal de Alfenas (UNIFAL-MG), Rua Gabriel Monteiro da Silva, n.700, Alfenas, MG CEP 37130-000, Brazil; 3 Instituto de Ciências Exatas, Universidade Federal de Alfenas(UNIFAL-MG), Rua Gabriel Monteiro da Silva, n.700, Alfenas, MG CEP 37130-000, Brazil; 4 Instituto de Ciências da Natureza, Universidade Federal de Alfenas (UNIFAL-MG), Rua Gabriel Monteiro da Silva, n.700, Alfenas, MG CEP 37130-000, Brazil; Kerala Forest Research Institute, INDIA

## Abstract

Species homogenization or floristic differentiation are two possible consequences of the fragmentation process in plant communities. Despite the few studies, it seems clear that fragments with low forest cover inserted in anthropogenic matrices are more likely to experience floristic homogenization. However, the homogenization process has two other components, genetic and functional, which have not been investigated. The purpose of this study was to verify whether there was homogenization of tree reproductive functions in a fragmented landscape and, if found, to determine how the process was influenced by landscape composition. The study was conducted in eight fragments in southwest Brazil. The study was conducted in eight fragments in southwestern Brazil. In each fragment, all individual trees were sampled that had a diameter at breast height ≥3 cm, in ten plots (0.2 ha) and, classified within 26 reproductive functional types (RFTs). The process of functional homogenization was evaluated using additive partitioning of diversity. Additionally, the effect of landscape composition on functional diversity and on the number of individuals within each RFT was evaluated using a generalized linear mixed model. appeared to be in a process of functional homogenization (dominance of RFTs, alpha diversity lower than expected by chance and and low beta diversity). More than 50% of the RFTs and the functional diversity were affected by the landscape parameters. In general, the percentage of forest cover has a positive effect on RFTs while the percentage of coffee matrix has a negative one. The process of functional homogenization has serious consequences for biodiversity conservation because some functions may disappear that, in the long term, would threaten the fragments. This study contributes to a better understanding of how landscape changes affect the functional diversity, abundance of individuals in RFTs and the process of functional homogenization, as well as how to manage fragmented landscapes.

## Introduction

One of the major threats to tropical biodiversity is the fragmentation process, which can cause changes in the structure, composition and functions of communities over time [1]. In plant communities, species homogenization and floristic differentiation are two possible consequences of this process [2,3]. Species homogenization is when sensitive species disappear locally and fragments are dominated by tolerant species [4]. This process occurs in fragments within the same modified human landscape that have similar abiotic and biotic pressures (environmental conditions, matrix type). However, floristic differentiation may occur in fragments under different environmental conditions and/or located in a heterogeneous landscape. In this scenario, the initial floristic differences may be amplified over time due to diverging environmental conditions caused by disturbance or other factors [4].

Some studies have shown flora homogenization in fragments [2], while others have indicated that this phenomenon is not always predominant, and that small fragments located in modified human landscapes can retain high levels of biodiversity [3,5]. A possible explanation for these different results is that, over the time, landscape composition (matrix type and forest cover) determines the changes in species of a plant assemblage in fragments that occur in human modified landscapes [2,3,4,5].

A reduction in forest cover and the consequent loss of habitat heterogeneity in fragmented landscapes may diminish the population size of forest species [6] and the number of species in a site [7]. Moreover, anthropogenic matrices can present different negative effects on remnant communities in forest fragments, depending on the characteristics of the matrix and the intensity of land use [8,9]. The matrix could become a strong barrier to some animal species, if its vegetation is structurally different from the original (natural) matrix. In addition, agrochemicals used in these environments have been associated with a reduction in plant richness [10,11] and the disappearance of pollinating insects, which alters mutualistic relationships [12,13]. Thus, landscapes with low forest cover commonly present in anthropogenic matrices are more likely to be experiencing a floristic homogenization process. The few studies about floristic homogenization have shown that the process is dependent on landscape composition [2,3]. However, the homogenization process has two other components, genetic and functional, which have not been investigated. Functional diversity refers to the range and value of species and organism traits that influence the functioning of an ecosystem [14] and it is an important property for maintaining the function and resilience of a community [15]. The functional homogenization process is similar to floristic homogenization. It occurs when communities lose more specific functions and become dominated by generalist functions, which leads to a reduction in functional diversity. However, a reduction in functional diversity does not necessarily mean a reduction in species diversity [16]. In other words, a fragment may have a large number of species performing the same ecological functions. In this situation, despite the high diversity of species, there is low functional diversity [16].

Studying functional diversity has greater biological significance than only considering richness and diversity of species because it connects individuals to their responses to environmental conditions [17]. The local extinction of a species does not necessarily mean the loss of its ecological function, since other species or individuals may perform the same function. Therefore, extinction of many species performing different functions will threaten an ecosystem more than the extinction of species that perform the same role. However, even if the extinction of redundant species occurs, the community's resilience to perturbations could still be altered [18].

Although no studies have investigated the functional homogenization process in plant communities, some studies have shown a reduction of more sensitive reproductive functions in fragmented environments, such as self-incompatible reproductive systems and pollination by vertebrates [19, 20]. These works showed that a modification in edge microclimate [20] and a decrease in fragment area [19] can reduce functional diversity. For this reason, we believe that functional homogenization is also dependent on landscape context. Based on this, the purpose of the present study was to verify the following: (i) whether there was homogenization of tree reproductive functions, a change in functional diversity, and dominance of individuals within a few reproductive functional types (RFTs) in fragmented landscapes and (ii) how landscape composition affects functional diversity and abundance of individuals within RFTs. To calculate functional diversity, different aspects can be considered, for example, physiological characteristics and succession class. This study used reproductive characteristics because they are related to the maintenance of an assemblage over time [19]. The study did not use information about succession class because a previous study of the same species [21] revealed that about 75% of the taxa do not provide reliable (based on experiments on irradiation influence on seed germination and seedling growth) data that allows them to be classified as pioneer and climax species. We expected the studied fragments would show a process of reproductive functional homogenisation since they are located in a landscape with low forest cover (<9%) and surrounded by anthropogenic matrices. We expected the studied fragments would show a process of reproductive functional homogenization because they are located in a landscape with low forest cover (<9%) and are surrounded by anthropogenic matrices. We predicted that landscapes with a greater proportion of pasture and sugarcane matrices (inhospitable matrices) would show a reduction in both functional diversity and abundance of individuals within more sensitive RFTs, such as those involving plant-animal interactions (animal pollination and dispersal, and self incompatible reproductive systems) [22]. On the other hand, we envisaged that the abundance within sensitive functional groups would increase in landscapes with a greater percentage of forest cover or coffee matrix (a shrubby, perennial matrix that is more similar to the fragments in terms of structure [3]. This information is highly relevant in the context of fragmentation, since the management of forest fragments in human modified landscapes requires understanding how land use changes affect remnant communities. In addition, this study contributes to understanding the biota homogenization process and forest fragmentation.

## Methods

### Study area

**Fig 1:**
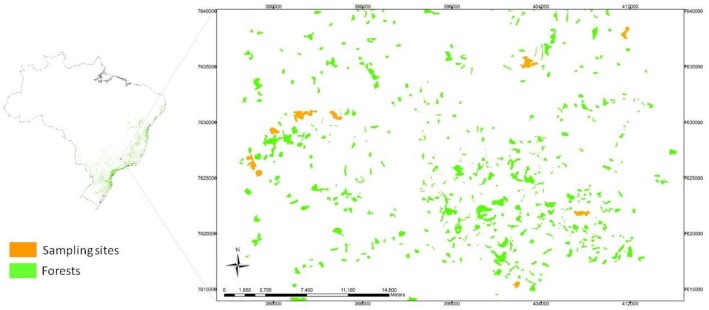
Location of the eight fragments studied in Alfenas, Minas Gerais. Orange polygons represent studied forest and green polygons represent others forests patches.

The study was conducted in eight forest fragments in Alfenas, Minas Gerais, Brazil (Table 1) (Fig 1). The areas are on private land and the farmers gave permission to conduct the study on their sites. The distance between fragments ranged from 3.1 to 49.6 km (mean: 28.1 ± 14.8 km). According to the Köppen-Geiger system, the climate is classified as Cwb (dry winters and temperate summers), the average temperature is 16.9°C in the winter and 21.5°C in the summer, and the average precipitation is 26 mm in the winter and 290 mm in the summer (1500 mm annually) [23]. The region has a predominantly hilly relief with elevations ranging from 720 to 1350 m.

**Table 1:** Fragment location, fragment area and values of landscape parameters analyzed, area of forest cover, percentage of COVER (forest cover) and percentage of matrices around fragments.

Fragment	Location	Area (ha)	Area of Forest cover (ha)	% COVER	% Coffee	% Sugarcane	% Pasture
**1**	21°29'13.13"S	20.91	42.04	13.39	15.72	31.39	33.14
	46°5'40.32"W						
**2**	21°26'14.51"S	22.99	29.45	9.38	51.83	0.00	14.02
	46°8'46.93"W						
**3**	21°34'42.37"S	28.57	77.05	24.54	16.83	45.13	10.5
	45°58'15.04"W						
**4**	21°33' 44.68"S	36.85	36.83	11.73	62.87	0.00	13.74
	45°56'12.80"W						
**5**	21°27'50.38"S	37.05	42.89	13.66	0.00	58.33	0.00
	45°54'58.10"W						
**6**	21°25'25.97"S	56.05	81.57	25.98	21.19	36.96	8.18
	46°5'8.03"W						
**7**	21°28'16.28"S	81.55	82.99	26.43	1.17	0.00	51.56
	46°7'22.43"W						
**8**	21°25'27.26"S	87.18	87.33	27.75	0.00	32.51	2.3
	46°9'35.66"W						

The fragments studied are preserved remnants of submontane semideciduous Atlantic Forest. The most dominant families of trees in the studied fragments are Fabaceae, Myrtaceae, Lauraceae, Meliaceae, and Euphorbiaceae. The most dominant tree species are *Copaifera langsdorffii* Desf., *Ocotea odorífera* (Vell.) Rohwer., *Cryptocarya aschersoniana* Mez., *Metrodorea stipularis* Mart., and *Miconia willdenowii* Klotzsch ex Naudin. This study did not involve endangered or protected species. The region only has about 9% remnant native forest and the most common matrix types are pastures (51%), perennial (mainly coffee—17%) and annual crops (mainly sugarcane and corn—7%) [24]. The region excelled as a coffee producer in the 19th and 20th centuries; however, it was also part of a traditional agropastoral system in the 18th century and early 19th century [25]. Therefore, forest fragmentation in the region started at this time.

We selected the fragments by classifying remnant forest areas using images (20 m resolution) from 2009 that were taken by the Sino-Brazilian CBERS-2B satellite [26]. In order to select the fragments, the following criteria were applied: i) similarity of degradation by observing spectral attributes, such as color and texture; ii) a minimum distance of four kilometers between areas, to ensure sample independence; and iii) a central fragment size between 15 ha and 100 ha.

A control area for was not used for two reasons: (i) the nearest continuous forest is Serra do Mar State Park, which is approximately 300 km from the study area and has a vegetation classified as ombrophilous forest, not semideciduous forest; and (ii) a control area is not essential to address the study questions because the goal of the study was to verify the homogenization process in fragmented areas and see how landscape composition affects functional diversity (not to compare fragmented areas with continuous areas).

### Species and individual sampling and landscape composition

All trees with a DBH (diameter at breast height) ≥3 cm in ten plots of 10 x 20 m (0.2 ha per fragment) were sampled in each of the eight studied fragments (1.6 ha total). The criterion of 3 cm was used to sample a larger number of species and individuals; 5 cm, which is more commonly used, can lead to a reduction in sampling by up to 60% (Martins & Santos 1999). The plots were randomly selected between 10 m from the edge of the center of the fragments in order to obtain the maximum habitat heterogeneity in the forest fragment and reproductive functional richness of the trees. All individuals sampled (2,018) were classified within 26 reproductive functional types (RFTs) belonging to the following 7 functional categories: pollination type, floral and fruit rewards, floral and fruit size, dispersal syndromes, and sexual and reproductive systems (Table 2) [*sensu* 19]. RFT classification was based on the literature and field observations [*sensu* 19]. The classification of RFT was based on the literature data and field observations [*sensu* 19]. Individuals with the same reproductive characteristics were considered to belong to the same RFT; for example, individuals dispersed by animals were considered as belonging to the zoochorous dispersal RFT. The abundance of individuals within each RFT for each landscape was the sum of the values from the ten plots sampled.

**Table 2:** Functional categories studied with their respective reproductive functional types (RFTs).

Functional Categories	Reproductive Functional Types (RFTs)
**Pollination Systems**	Bees
	Beetles
	Flies
	Generalist insects
	Vertebrates
	Wind
**Floral reward**	Floral tissues
	Nectar
	Nectar/Pollen
	Odor
	Oil
	Pollen
	Shelter
	Without resource
**Flower size**	Small (< 1 cm)
	Large (> 1 cm)
**Dispersal syndromes**	Anemochory
	Autochory
	Zoochory
**Fruit reward**	Aril
	Pulp
	Without resource
**Fruit size**	Small (< 1 cm)
	Large (> 1 cm)
**Reproductive systems**	Self compatible
	Self incompatible

A circle with a radius of 1,000m was drawn from the center of each fragment (buffer of 1,000 m) and the percentage of each landscape composition (COVER and MATRIX) was calculated using the programs ArcGIS (Version 10.0, ESRI) and Fragstats programs [27]. COVER is the proportion of forest in the buffer, and was calculated using the formula: *Af* = (π*r ²*PF)/100, where *Af* is the total area of forests (forest), *πr ²* is the area of the buffer around the central fragment, and *PF* is the total proportion of forest in radius *r* of each buffer. MATRIX (Landscape index) quantifies the proportion of buffer covered by each habitat matrix (coffee, pasture, and sugarcane), and was calculated using the equation: *Am* = (πr²*PM)/100, where *Am* is the sum of anthropogenic habitat matrix areas and *PM* is the total proportion of habitat matrix in each buffer. All the landscape metrics were log10 transformed.

We selected this buffer size because the landscape composition affects animal pollinators and seed dispersers for larger distances which can influence mutualistic relationships between plants and animals and consequently the RFTs. Dispersers, such as bats [28], and pollinators, such as Euglossine bees [29], are known to cover long distances (flights), even in fragmented landscapes.

### Functional diversity calculation and statistical analysis

To calculate the functional diversity of the landscape, a method described by Petchey and Gaston (2002) [30], called FD (functional diversity), was used. FD measures the extent of complementary between values of individual traits. Greater differences between these values represent greater complementary and, therefore, a higher FD. An FD value was obtained for each fragment. The FD calculation is based on a cluster analysis and has the following four steps: (I) creating a functional matrix (abundance of individuals × functional traits) for each fragment studied; (II) converting the functional matrix into a distance matrix; (III) producing a dendrogram from the distance matrix; and (IV) calculating the total length of the dendrogram branches. The distance measure used was the Gower distance [31]. There are other functional diversity measures [32]; however, FD better explains the community functional differences because it is not affected by species number [17]. The analyses were performed with the software R (R Development Core Team) *2*.*5*, and the FD, ade4, and picante packages. The homogenization process was evaluated using additive partitioning of diversity. Areas with low alpha (within fragment) and beta (between fragments) diversity are considered homogenized [3] and areas with high alpha and beta diversity are in a floristic differentiation process [2]. In the additive partitioning of species diversity, the diversity of RFTs of the region or the total (γ) is split into alpha and beta components that are expressed in the same units. The total RFT diversity (γ) for a set of samples is split into the RFT averages present in the samples (alpha) and the RFT averages absent in each sample (beta). Therefore, γ = alpha+ beta 1+ beta 2+…beta n, where n = number of levels, and beta n = alpha n+1- alpha n [31]. The average diversity from plots (beta 1) and fragments (beta 2) were calculated as a component of total diversity in order to verify the spatial variation of diversity. This observed diversity was compared with a null distribution model of diversity consisting of the mean diversity obtained through 1000 randomizations. The 1000 randomizations create the expected diversity if the distribution of individuals is random. This analysis also allowed us to compare the results observed to what would be expected if the distribution was random, that is, when there is no factor affecting the community structure [33]. A difference from the expected values may indicate dispersal limitation, environmental heterogeneity and/or effect of landscape on the observed diversity [33]. The expected diversity was calculated with the software Partition [33]. To calculate the diversity of the RFTs, the decomposition of Hill numbers (1973) with q = 1 was used, which is an approach proposed by Jost (2007) [34] that indicates the effective number of species that a community would have if all species had a weight proportional to their relative abundances (exp(H')).

In order to verify whether the beta diversity observed was due to loss of ordinate functions (nestedness) or exchange functions (turnover), we decomposed the beta diversity of these two components. The index of beta diversity ranges from 0 (completely similar) to 1 (completely dissimilar). Subsequently, we partitioned the total dissimilarities (B_SOR_) in the proportion generated by nestedness (B_NES_) and in the proportion generated by turnover (B_SIM_), where B_SOR_ = B_NES_ + B_SIM_ [35]. These analyses were carried out in R (R Development Core Team 2007) using the functions “beta-multi. R” in the package “betaparte.” A recent study [36] showed that in this method the nestedness-resultant component accounts for only richness differences derived from nested patterns, while in other methods the richness difference dissimilarity accounts for all kinds of richness differences. Moreover, in the method proposed by Baselga (2010) [35] the replacement component is independent of richness difference.

In order to describe the communities in terms of composition and check for dominant RFTs within each functional category, ANOVA and Tukey’s test were conducted when there were more than two RFTs within the same functional category, and a T-test was conducted when there were only to RFTs. This analysis helps us verify, for example, if there is a dominance of RFT characteristics from more generalist species, such as pioneer trees, small fruits and self compatibility. The similarity between the fragments was analyzed using the Bray-Curtis index. This index was selected because it considers the individuals in RFT abundance [37].

The landscape composition (COVER, percentage of coffee, pasture and sugar cane) effect on functional diversity (FD) and on number of individuals within RFTs was evaluated using a generalized linear mixed model (glmer in R package lme4) and included the fragments as a random factor. The fragment was used as a random variable because it was not in our interest check the fragment effect on functional diversity (FD) and on number of individuals within RFTs. In other words, we took into account the samples dependence within each fragment and increased the test power by subtraction of the random factor error variation. A generalised linear mixed model was run to model the (i) abundance within RFTs (response data), with Poisson distribution, and (ii) functional diversity (response data), with Guassian distribution. The effect of isolated landscape parameters (independent variable) and the effect of parameters in pairs was checked through an additive relationship. An additive relationship means that the value of a variable does not depend (there is no interaction) on the value of another variable, but the variables are necessary for a good model fit and a more accurate estimation. We did not analyze the effect of the fragment area, nor the size of forest cover on functional diversity (FD) and on the number of individuals within RFTs, because these landscape parameters were correlated with COVER.

Significant models were validated by normality and independence testing of residue. To assess the performance of models, the Akaike Information Criterion (AIC) was employed [38]. The best model was based on the lowest AIC value [33], the ΔAIC and Akaike weights (wAIC). The ΔAIC is the difference between the model and the minimum AIC values, and the models with ΔAIC< 2 are considered as having substantial support, while the Akaike weights (wAIC) describe the probability of the model being the best model among those studied [38]. Only the results from valid models are presented.

## Results

**Fig 2:**
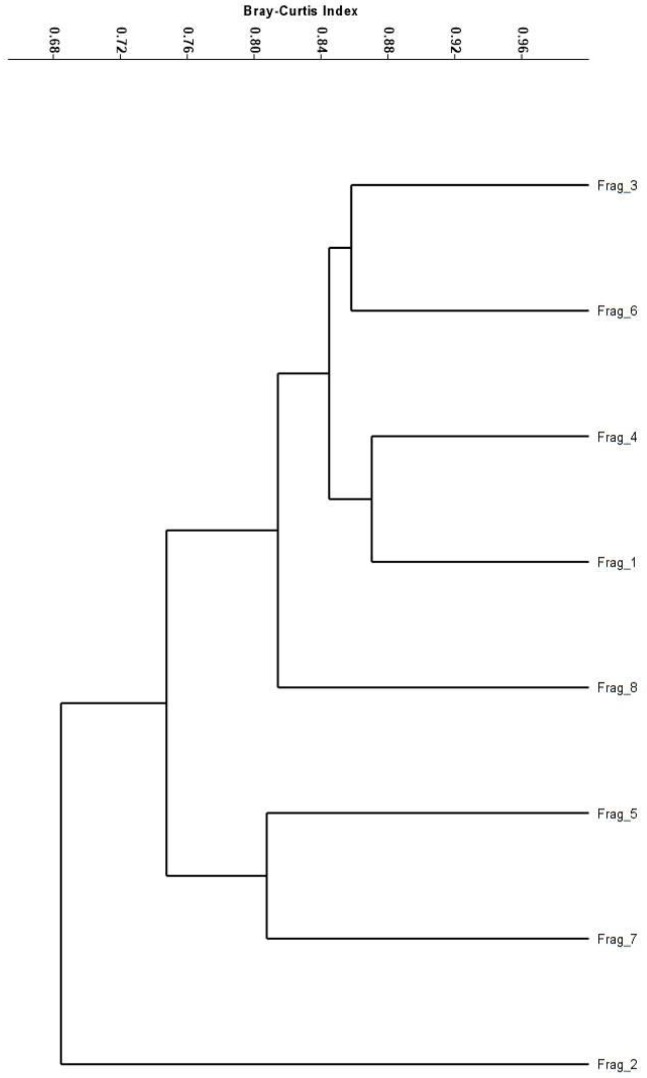
Functional similarity. Dendrogram of functional similarity (Bray-Curtis index) produced by a cluster analysis (UPGMA connection method) of composition of reproductive functional types (RFTs) among the eight fragments.

The study sampled 2,018 individuals belonging to 184 plant species in the eight fragments As expected, there were some dominant RFTs (i.e., most of the individuals were concentrated in a few RFTs). The RFTs with a greater number of individuals, within each functional category, were the following: pollinated by bees (35.2%), generalist insects (27.5%) (F_7;64_ = 43.3, p = 0.0001), floral nectar as reward (25.9%) (F_6;49_ = 17.8, p = 0.0001), small flowers (55.1%) (t_8_ = 9.9,p = 0.001), zoochorous dispersal (72.9%) (F_2;21_ = 102.2, p = 0.0001), fruit with pulp (45.5%) (F_2;21_ = 20.3, p = 0.0001), and self compatible reproductive system (31.3%) (t_8_ = 2.7, p = 0.01). Consequently, the individual abundances within RFTs among the fragments were very similar (Bray-Curtis index from 0.68 to 0.89) (Fig 2).

The individual dominance in a few RFTs was reflected in the alpha and beta functional diversity (FD) of the fragments. The functional diversity ranged from 0.35 to 0.58, and more than 60% of the fragments presented an FD lower than 0.5.

**Fig 3:**
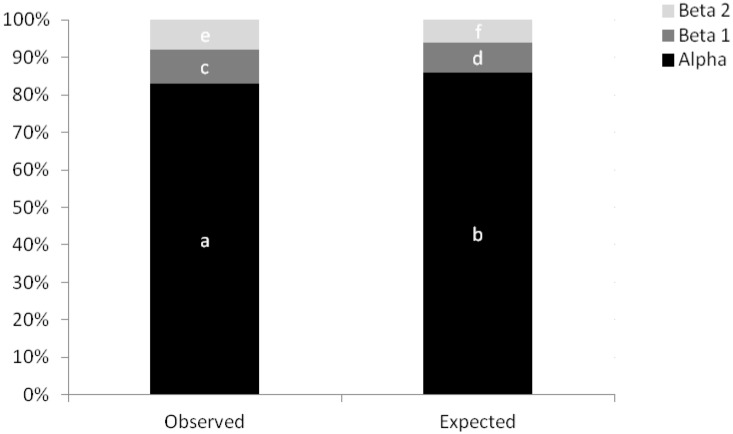
Additive partition of functional diversity. Additive partition of functional diversity for reproductive functional types (RFTs) using q = 1 (all RFTs have a weight proportional to their relative abundances). Alpha is the average diversity within the plots. Beta is the average diversity absent in the plots. Beta2 is the average diversity absent in the fragments. The sum of the alpha and beta components results in the gamma diversity of each fragment. The sum of alpha, beta, and beta2 components results in the gamma diversity of the region. Different letters indicate observed values significantly different than the expected value if the distribution were random.

Alpha diversity (inside plots) represented 82% of the gamma diversity. However, this diversity was lower than expected by chance (p<0.001), indicating that some factor is decreasing diversity in the fragments. The beta 1 diversity (between plots) was 9%, and the beta diversity 2 (between fragments) was 8%, suggesting that the fragments are in a process of functional homogenization (Fig 3). The decomposition analysis revealed that 68% of the beta diversity is due to nestedness, in other words, there is an ordered loss of reproductive functions. The less diverse fragments are a subset of the more diverse fragments.

As expected, the landscape parameters affected both the functional diversity and the number of individuals within the RFTs; 52% of the RFTs were affected. The landscape parameters with the greatest influence were the percentage of forest cover into the buffer (15% of RFTs) and the additive relationship of this parameter with the percentage of coffee matrix; together, 32% of the RFTs were affected.

The pollen and odour (floral rewards), large flowers, and self compatible reproductive system RFTs benefitted from an increase in percentage of forest cover. The additive relationship of percentage of forest cover with percentage of coffee matrix was antagonistic. When the forest cover value is constant, an increase in the percentage of the coffee matrix around the forest fragment results in a negative effect on the following RFTs: pollination by beetles, floral tissues as reward, small fruits, and self incompatible reproductive system. On the other hand, when the percentage of the coffee matrix is constant, an increase in percentage of forest cover has a positive effect on these RFTs. Additionally, an increase in percentage of sugarcane reduces the functional diversity of the fragment, and increases the number of trees in the anemochoric dispersal and pollination by flies RFTs. Furthermore, an increase in percentage of the pasture matrix increases the trees within the pollination by vertebrates and shelter and nectar (floral rewards) RFTs (Table 3).

**Table 3:** Generalized linear mixed model.

RFTs	Models			
**Functional diversity**	Estimate	p value	ΔAICc	w
***Fixed effects***				
**Intercept**	0.15	0.001	0.0	0.95
**Sugarcane**	- 0.02	0.03		
***Random effect***				
**Fragment**	0.00001	-	-	-
**Residuals**	0.06	-	-	-
**Beetles**	Estimate	p value	ΔAICc	w
***Fixed effects***				
**Intercept**	9.56	0.002	0.0	0.91
**Cover**	7.21	0.002		
**Coffee**	- 0.81	0.001		
***Random effect***				
**Fragment**	0.02	-	-	-
**Residuals**	0.15	-	-	-
**Flies**	Estimate	p value	ΔAICc	w
***Fixed effects***				
**Intercept**	1.30	< 0.05	0.0	0.45
**Sugarcane**	0.31	0.0001		
***Random effect***				
**Fragment**	0	-	-	-
**Residuals**	0	-	-	-
**Vertebrates**	Estimate	p value	ΔAICc	w
***Fixed effects***				
**Intercept**	1.67	0.27	0.0	0.73
**Pasture**	1.23	0.002		
***Random effect***				
**Fragment**	0.19	-	-	-
**Residuals**	0.08	-	-	-
**Pollen**	Estimate	p value	ΔAICc	w
***Fixed effects***				
**Intercept**	5.21	0.004	0.0	0.49
**Cover**	3.96	0.003		
***Random effect***				
**Fragment**	0.17	-	-	-
**Residuals**	0.41	-	-	-
**Shelter**	Estimate	p value	ΔAICc	w
***Fixed effects***				
**Intercept**	- 2.82	0.0003	0.0	0.56
**Pasture**	2.04	0.002		
***Random effect***				
**Fragment**	0.41	-	-	-
**Residuals**	0.64	-	-	-
**Nectar**	Estimate	p value	ΔAICc	w
***Fixed effects***				
**Intercept**	1.27	< 0.05	0.0	0.42
**Pasture**	0.59	0.02		
***Random effect***				
**Fragment**	0.07	-	-	-
**Residuals**	0.28	-	-	-
**Odor**	Estimate	p value	ΔAICc	w
***Fixed effects***				
**Intercept**	8.36	0.001	0.0	0.37
**Cover**	6.02	0.003		
***Random effect***				
**Fragment**	0.39	-	-	-
**Residuals**	0.63	-	-	-
**Floral tissues**	Estimate	p value	ΔAICc	w
***Fixed effects***				
**Intercept**	9.49	0.05	-	-
**Cover**	6.02	0.03		
**Coffee**	- 0.95	0.04		
***Random effect***				
**Fragment**	0.46	-	-	-
**Residuals**	0.68	-	-	-
**Big flowers**	Estimate	p value	ΔAICc	w
***Fixed effects***				
**Intercept**	3.19	0.0004	-	-
**Cover**	1.51	0.02		
***Random effect***				
**Fragment**	0.04	-	-	-
**Residuals**	0.21	-	-	-
**Anemochory**	Estimate	p value	ΔAICc	w
***Fixed effects***				
**Intercept**	3.19	0.0004	0.0	0.61
**Sugarcane**	-0.57	0.0002		
***Random effect***				
**Fragment**	0	-	-	-
**Residuals**	0	-	-	-
**Small fruits**	Estimate	p value	ΔAICc	w
***Fixed effects***				
**Intercept**	3.19	< 0.05	0.0	0.61
**Cover**	2.43	< 0.05		
**Coffee**	-0.52	< 0.05		
***Random effect***				
**Fragment**	0.008	-	-	-
**Residuals**	0.09	-	-	-
**Self compatible**	Estimate	p value	ΔAICc	w
***Fixed effects***				
**Intercept**	5.63	< 0.05	0.0	0.48
**Cover**	2.54	0.008		
***Random effect***				
**Fragment**	0.09	-	-	-
**Residuals**	0.31	-	-	-
**Self incompatible**	Estimate	p value	ΔAICc	w
***Fixed effects***				
**Intercept**	3.52	< 0.05	-	-
**Cover**	1.29	0.04		
**Coffee**	-0.55	0.002		
***Random effect***				
**Fragment**	0.05	-	-	-
**Residuals**	0.23	-	-	-

Generalized linear mixed model relating the landscape parameters and individual abundance within RFTs and functional diversity. Fragment was used as a random effect. Models with Δ AICc > 2.0 were rejected and not included in the table. ΔAICc = Difference in AIC from one model to one with the lowest AIC value. w = AICc weight.

## Discussion

As expected, the fragments seem to be in a homogenization process. The alpha diversity is less than expected by change and the beta diversity is very low, less 10% of the gamma diversity. In fact, most of the trees are concentrated for the same few dominant RFTs in almost all remnants, 68% of the beta diversity is due to nestedness and the fragments are very similar in their functional composition. The landscape parameters analysed affected the functional diversity of more than 50% of RFTs. In general, an increase in percentage of forest cover presented a positive effect on RFTs, while the percentage of the coffee matrix exhibited a negative one. In addition, the functional diversity as the increasing percentage of sugarcane increases.

This present study has a sample limitation of: only 8 forest fragments. However, besides this, we think that the results of work could be important. This is the first study to attest functional diversity homogenization in a fragmented landscape. Four aspects of our analysis suggest that the remnants studied are experiencing this process: (i) in general, the RFTs with more individuals (dominants) are characteristics of generalist species; (ii) alpha diversity is lower than expected by change; (iii)the RFTs of the tree assemblage are very similar among the fragments(low beta diversity), and (iv) the beta diversity process is due to nestedness.

All fragments presented all functional groups, but the number of individuals within the pollinated by bees and generalist insects, nectar as floral reward, small flowers, zoochorous dispersial, fruit with pulp, and self compatible reproductive system RFTs were very high. The pollination by generalist insects, small flower and self compatible reproductive system RFGs are more tolerant groups, occurring in different environments. The European honey bee (*Apis mellifera*), an invasive species that has become abundant in Atlantic Forest areas [39,40], may be preventing the local extinction of trees with nectar as floral reward and pollinated by bees RFTs. In addition, generalist birds and small mammals, which are very common in the region [41], may be preventing the local extinction of trees with the zoochorous dispersal and fleshy fruits RFTs. The dominance of RFTs characteristic of generalist species may be an indication that the fragments are losing individuals from more sensitive functions and are becoming dominated by species with generalists functions, which is one of the characteristic of homogenization [2]. One aspect of the results that reinforces this hypothesis is that alpha diversity was lower than expected by chance. In other words, the loss of more sensitive functions and increase of more general functions is leading to functional homogenization and limiting the accumulation of functions in the fragments [2,42].

Low functional diversity may indicate two different problems: (i) the fragments do not have all of the reproductive functions or (ii) they present few individuals performing these functions [17,30,43]. Both problems have serious consequences for the long-term maintenance and stability of communities [44]. The loss of species that perform certain reproductive functions can also affect mutualistic relationships, leading to the disappearance of animal pollinators and seed dispersers [45,46,47]. The studied fragments presented low redundancy because there were a low number of individuals and species performing some functions, especially the more sensitive ones. Therefore, any disturbance is able to destabilize the plant community and lead to function loss. In other words, areas with low redundancy can have problems recovering from disturbances [43].

In addition to RFT dominance and lower expected alpha diversity, the fragments also showed low beta diversity and a pattern of nestedness. This is a strong indication of homogenization. The partition of diversity is recommended to check the homogenization process [4], and the few studies that have investigated the floristic homogenization process have shown that homogenized areas have low beta diversity, which is a major factor to determine the homogenization [2,42]. A nested pattern also indicates that there is orderly loss of functions, which means the fragments are losing the same functions from sensitive species and are being dominated by more functions from generalist species (small flowers, self compatible reproductive system) [48,49,50].

The few studies that have investigated floristic homogenization in tropical forests inferred that this process could be dependent on landscape context, where environments with mechanized agriculture and low forest cover are more likely to experience an ordered loss of species [2,35]. As expected, our results indicate that functional diversity and RFTs (over 50%) were affected by landscape parameters. All parameters measured affected at least one RFT, especially the additive relationship between forest cover and percentage of coffee matrix (32% of RGFs). As expected, forest cover had a significant and positive effect on several RFTs, even in a highly fragmented landscape, where the remnants were below the forest cover threshold (ranging from 9 to 27%). Various studies report that greater forest cover helps maintain species and populations, as well as all functions of an ecosystem, since landscapes with greater forest cover provide resources and shelter for the organisms [51,52]. Also, as expected, this parameter favored the abundance of individuals with more sensitive and specialized reproductive functions, such as large fruits and odor as a floral reward.

In relation to the anthropogenic matrices, with the exception of sugar cane that had a negative effect, the results are contrary to expectations. The coffee plantation resulted in a negative effect on RFTs, especially the most sensitive ones, such as pollination by beetles and self incompatible reproductive system. This was an unexpected result because we thought this matrix would have a positive effect, since it is more structurally similar (plant density and height) to the forest fragments and therefore, could provide resources and shelter for animals and soften the edge effect [3]. Our hypothesis for this unexpected result is that the management practices in these matrices could have more of an impact on RFTs than the matrix type. In coffee plantations large amounts of fertilizer and pesticide are applied annually [53]. The use of these products has been linked to the death of pollinating insects [54,55], altered biogeochemical cycles [56], and a reduction in plant richness [57]. Additionally, the mechanized harvesting of coffee has also been associated with a reduction in plant richness [58]. We believe, therefore, that despite the apparent advantages of this crop to biota (by decreasing the edge effect due to the more shrubby habit of the plants), management practices of coffee plantations are a serious threat to RFTs. A survey carried out in Sweden showed that less managed landscapes have higher species richness and can maintain the species pool on a regional scale [58]. Another point that reinforces our hypothesis is that the pasture matrix, which comprises open areas that amplify the edge effect and make it difficult for organisms to disperse, had a positive effect on some RFTs. This is probably because there are few or no harmful management practices, with little or no use of pesticides, machines or burning, in this matrix in the study area.

Our study also revealed that fragments located in sites with a larger percentage of sugarcane presented lower functional diversity. We attribute this result to the aggressive management of these crops, because large amounts of pesticides and burning are regularly used [59]. Fire can invade the fragments and kill species, leading to a loss of reproductive functions and/or death and loss of different animals as well as change the mutualistic relationships [60]. This situation could worsen in the future, both in Brazil and other countries, such as the United States, India and China, where there has been an enormous increase in sugarcane plantations due to the demand for ethanol and sugar [61]. For example, in Brazil, an additional eight million hectares are destined for sugarcane production [62, 63]. From 1995 to 2011, the area of sugarcane cultivation increased by 300% and 130% in the states of Minas Gerais and São Paulo, respectively, two of the most populated Brazilian states [63].

## Conclusions

This study is the first to show that landscape composition has a very important role in plant functional homogenization in forest remnants, affecting both functional diversity and abundance of individuals in RFTs. Functional homogenization (low beta diversity) has serious consequences for biodiversity conservation, since some functions, which are becoming rarer, may disappear altogether and threaten the long-term success of fragments [64,65,66]. This study also indicates that forest cover loss and an increasing percentage of farm crops in the region, mainly coffee, seems to be responsible for reproductive function homogenization. In addition, the study contributes to a better understanding of how the current landscape changes affect the functional diversity, abundance of individuals in RFTs, and the process of functional homogenization. This knowledge could be useful for large-scale ecological predictions because functional groups aggregate plants that respond similarly to disturbances, independent of the scale [67,68,69].

### Implications for conservation

The fragments are threatened, and although this study included only a small sample (eight fragments), we believe that other Atlantic Forest fragments are also undergoing a functional homogenization process because they have the same characteristics (small, isolated, inserted into modified human landscapes with little forest cover) [70]. Recovering diversity and functional redundancy in fragmented areas is necessary for perfect functioning of ecosystems. Without this, it is possible that over time the tree assembly in forest fragments will become functionally simplified.

This study could support actions to prevent further loss of reproductive function in the tree assembly, which could decrease and/or interrupt the anthropogenic impact. For example, (a) discouraging or prohibiting pesticide use in coffee plantations near the fragments via education programs offered to the producers, stricter laws and more effective controls; and (b) improving the conditions of the matrices, and reducing management, mechanization and fire. In addition, actions related to recovering lost reproductive functions could prevent further loss of reproductive function. For example, (a) planting tree species from the most endangered and/or lost reproductive functions, such as self-incompatible trees and those pollinated by vertebrates, and (b) improving the movement of seed and pollen dispersal by animals through ecological corridors and/or more permeable matrices.

Environmental recovery and conservation programs need to consider landscape composition. Landscape composition has a very strong relationship with functional diversity and the functional homogenization process. The management of cultivated areas around the forest remnants should be less aggressive (e.g., less mechanization and pesticides) or these conservations programs will not succeed.

## Supporting Information

S1 Data (XLSX)
